# Bright future or blind alley? CAR-T cell therapy for solid tumors

**DOI:** 10.3389/fimmu.2023.1045024

**Published:** 2023-01-24

**Authors:** Kai Zhang, Hong Chen, Fuqiang Li, Sheng Huang, Fei Chen, Yi Li

**Affiliations:** ^1^ Department of Oncology, 920th Hospital of Joint Logistics Support Force, Kunming, Yunnan, China; ^2^ Graduate School, Kunming Medical University, Kunming, Yunnan, China; ^3^ Department of Traditional Chinese Medicine, 920th Hospital of Joint Logistics Support Force, Kunming, Yunnan, China; ^4^ Department of Breast Surgery, Breast Cancer Center of the Third Affiliated Hospital of Kunming Medical University, Yunnan Cancer Hospital, Kunming, Yunnan, China; ^5^ Department of Medical Oncology, The Second Affiliated Hospital of Kunming Medical University, Kunming, Yunnan, China

**Keywords:** CAR-T cells, solid tumor, adoptive immunotherapy, tumor microenvironment, cytokine release syndrome, tumor infiltration, immune evasion

## Abstract

Chimeric antigen receptor (CAR) T cells therapy has emerged as a significant breakthrough in adoptive immunotherapy for hematological malignancies with FDA approval. However, the application of CAR-T cell therapy in solid tumors remains challenging, mostly due to lack of suitable CAR-T target antigens, insufficient trafficking and extravasation to tumor sites, and limited CAR-T survival in the hostile tumor microenvironment (TME). Herein, we reviewed the development of CARs and the clinical trials in solid tumors. Meanwhile, a “key-and-lock” relationship was used to describe the recognition of tumor antigen via CAR T cells. Some strategies, including dual-targets and receptor system switches or filter, have been explored to help CAR T cells matching targets specifically and to minimize on-target/off-tumor toxicities in normal tissues. Furthermore, the complex TME restricts CAT T cells activity through dense extracellular matrix, suppressive immune cells and cytokines. Recent innovations in engineered CARs to shield the inhibitory signaling molecules were also discussed, which efficiently promote CAR T functions in terms of expansion and survival to overcome the hurdles in the TME of solid tumors.

## 1 Introduction

Chimeric antigen receptor (CAR) T-cell therapy represents a significant breakthrough in adoptive T-cell therapies for cancer. CAR is engineered synthetic receptors that redirects T cells to recognize the corresponding antigens in a major histocompatibility complex (MHC)-independent manner, thus activating anti-tumor responses. To date, CAR-T cell therapy has achieved tremendous successes in hematological malignancies (1). The CAR-T cell targeting the pan-B-cell marker CD19, which became the first gene-therapy product approved by US Food and Drug Administration (FDA), had achieved largely advance in patients with relapsed or refractory acute lymphoblastic leukemia and diffuse large B-cell lymphoma, with complete remission rates of up to 90%. However, despite extensive research, CAR-T cell therapy for solid tumors have shown limited antitumor activity in early phase clinical trial, where the unique challenges must be addressed as lack of suitable CAR-T target antigens, insufficient trafficking to tumor sites, and limited CAR-T survival in the immunosuppressive tumor microenvironment (TME), and life-threatening CAR-T cells-associated toxicities. Tumor cells can construct conditions conducive to their own survival by stimulating new blood vessels, while also recruiting and stimulating immunosuppressive cells to aggregate in the TME, thus employing their secreted inhibitory cytokines and extracellular matrix (ECM) to create an environment that facilitates immune escape (2). Actually, CAR-T cells, like tumor-infiltrating T cells, are subject to physical, chemical, and cellular barriers in the TME. Furthermore, excessive activation of CAR-T cells may increase the risk of immune-related adverse events and on-target/off-tumor effects. Importantly, identifying mechanisms underlying these hurdles is vital to improve CAR T function with promising safety. Currently, many strategies to engineer more powerful CAR T cells have been proposed to mitigate antigen heterogeneity, to augment CAR T cells infiltration, to overcome immunosuppressive signaling within the TME, and to reduce toxicities (3). Herein, we describe the development of CARs and the clinical trials of CAR-T therapy for solid tumor. We next discuss the challenges posed by tumor antigens and hostile TME as well as the toxic effects associated with CAR T therapy, and present recent innovations to overcome these obstacles in solid tumors.

## 2 Development of CARs

**Figure 1:**
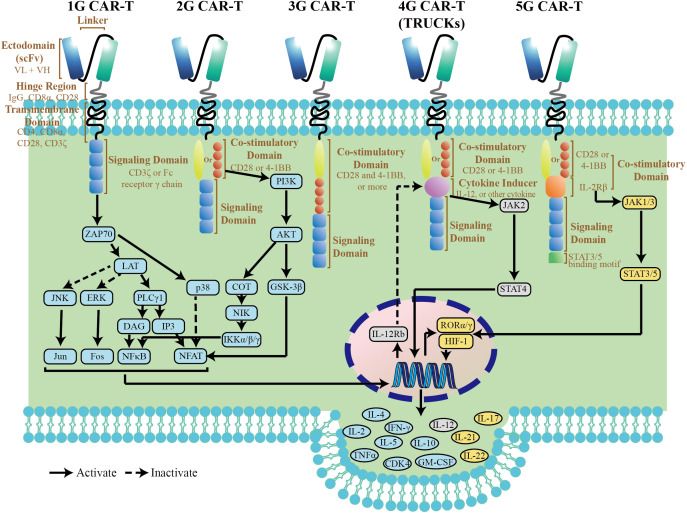
Structure diagram and simple pathway map of CAR-T cells. The pathway map is referenced from KEGG (https://www.genome.jp/kegg/).

The original intention of CARs is to avoid the restrictions of major histocompatibility complex (MHC) by specific immune cells. A CAR, an artificial fusion protein, is made of four key segments. The extracellular domain, typically derived from the single-chain variable fragment (scFv) of an antibody, is responsible for tumor antigen recognition (4). The hinge portion, located between scFv and transmembrane (TM) domain, provide flexibility to overcome steric hindrance, which is needed to allow the antigen domain to access the targeted epitope (5). Importantly, the hinge region with different length and composition can affect flexibility, CAR expression, signaling, and epitope recognition (6).The TM domain, along with the hinge region, anchors the CAR to cell membrane, which affects a downstream signaling cascade of T-cell activation. The intracellular signaling domain most commonly consists of a single CD3ζ domain and some co-stimulatory molecules. With a fast-paced journey to tackling the obstacles of CAR T therapy, five generations of CARs have been developed (**Figure 1**). The first generation CARs with only CD3ζ molecule in the intracellular signaling domain showed little therapeutic effects in early clinical trials (7). After that, co-stimulatory molecules, which enhance CAR T cell activity, were introduced into CARs, such as CD28 and CD137 (4-1BB). Depending on the number of co-stimulatory molecules, second (one domain) and third (two domains) generation CARs are designed, respectively (8). And most clinical trials are dominated by 2G and 3G CAR-T cells (9). However, the benefit of 3G CARs with two co-stimulatory molecules is model-dependent. Ramello reported that PSCA-specific second-generation CARs containing the CD28 transmembrane and co-stimulatory domains can induce additional sources of downstream signaling via the expression of a constitutively phosphorylated form of CD3ζ, suggesting that second-generation CARs could activate more intense signaling and superior antitumor efficacy as compared to third-generation CARs (10). To further optimize CAR function, some modifications of the intracellular domain were introduced based on the backbone of 2G CARs. The fourth generation CARs, also known as armored CARs or TRUCKS (T cells redirected for universal cytokine-mediated killing), are designated with a nuclear factor of activated T cells (NFAT) response element-driven cytokines, such as interleukin(IL)-12, IL-15, and granulocyte-macrophage colony-stimulating factor. Such TRUCKs, T cells redirected for universal cytokine-mediated killing have more improvement in anti-tumor efficacy and persistence than 2G CAR-T cells, which endows them with the potential to break through the immune suppressive microenvironment (11). The so-called fifth-generation (5G) CAR currently has been developed with an intracellular fragment of cytokine receptor (for instance, IL-2Rβ) and a STAT3-binding YXXQ motif. This new generation of CARs was capable of inducing cytokine signaling after triggering JAK-STAT3/5 pathway, which provided the T cells with superior cytolytic activity even after repeated antigen exposures (12).

## 3 Phase I/II clinical trials of CAR-T cell therapy against solid tumors

**Figure 2:**
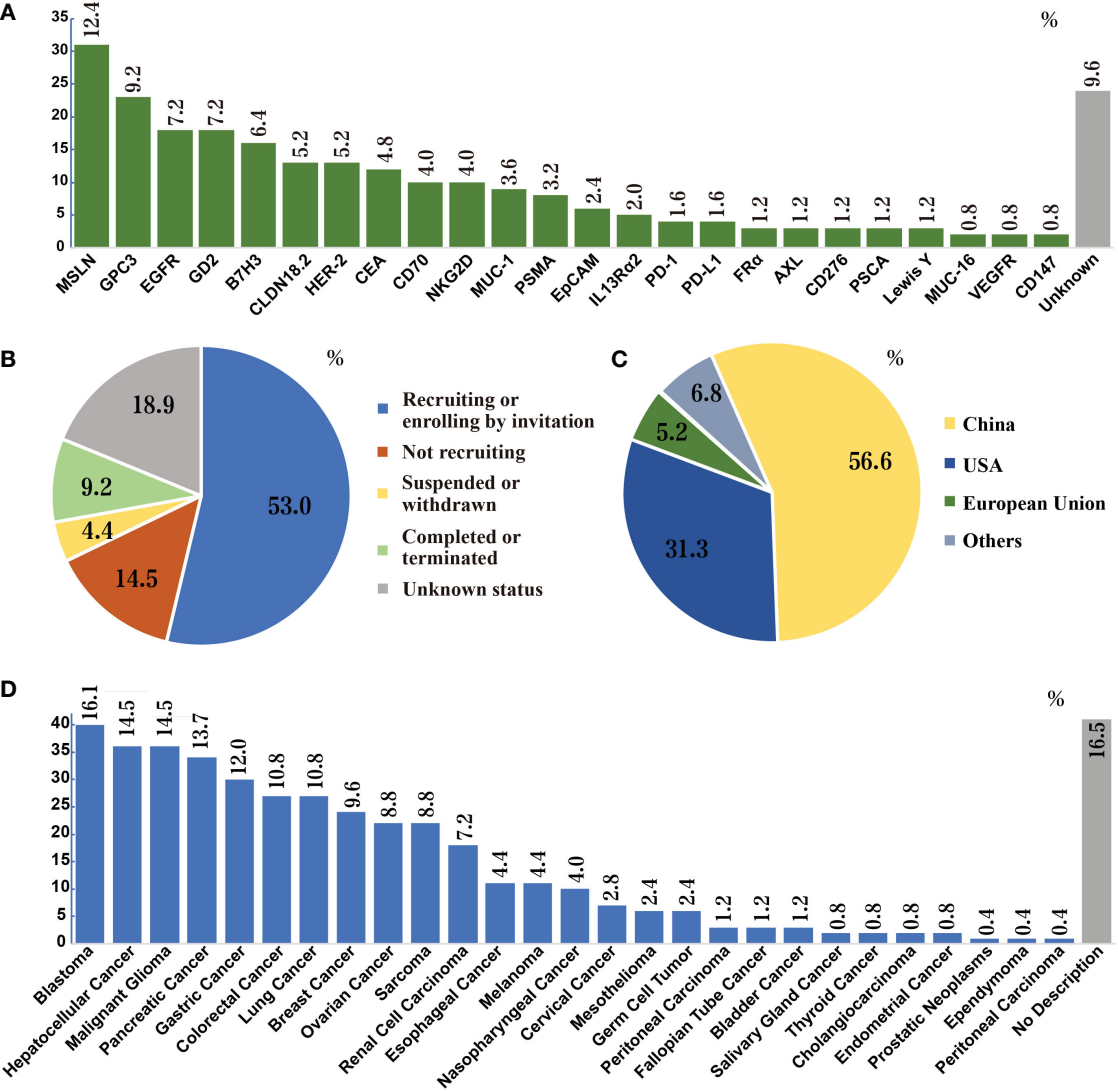
Search results of CAR-T cell therapies for solid tumors in NIH Clinical Trials Database (as of November 21, 2022). **(A)** Research frequencies of every target. **(B)** Status of clinical trials; over 60% of trials are ongoing. A total of 53 targets appeared in these clinical trials. The figure does not list the targets that appear only once (0.4%), which are: CD20, EphA2, CLDN6, CD40, TM4SF1, EpCAM, Nectin4/FAP, AFP, B4T2-001, ROR2, U87, ALPP, CD52, CD22, IL17Rα, CAIX, IM83, E2, IM92, CD171, IM96, ROR1, KKLC1, TAG72, CD56, CD33, MMP2, ICAM1 and gp100. **(C)** Countries or regions where the trials are located. The most clinical trials of CAR-T cell therapies for solid tumors are conducted in China, followed by the U.S. **(D)** Species of solid tumors studied in the clinical trials. Liver cancer, pancreatic cancer, breast cancer, sarcoma, and lung cancer are the five most studied solid tumors in clinical trials.

CAR T cell therapy has impressive achievements in the arena of hematological malignancies, in which seven CAR T products have been approved by the FDA (13), starting from the first CAR-T drug Kymriah^®^ (tisagenlecleucel, Novartis) in August 2017 for acute B lymphocytic leukemia (14) to most recently CARVYKTI™ (ciltacabtagene autoleucel, Legend Biotech) for CD19-positive relapsed or refractory diffuse large B-cell lymphoma in February 2022 (15). With the success of CAR T cells in hematologic malignancies, a growing number of clinical trials are underway focusing on translating this therapy to solid tumors. Up to date, 249 clinical trials of CAR T in solid tumors have been registered in the National Institutes of Health (NIH) database. All studies are phase I/II trails, and more than 50 potential targets have been investigated, including glypican-3 (GPC3; NCT02395250, NCT03146234), GD2 (NCT02761915), epidermal growth factor receptor (EGFR; NCT01869166), mesothelin (MSLN, NCT03054298, NCT03323944), mucin 1 (MUC-1, CD227; NCT02587689), human epidermal growth factor receptor-2 (HER2; NCT01935843), prostate-specific membrane antigen (PSMA; NCT03089203), carcinoembryonic antigen (CEA; NCT02349724), and claudin18.2 (CLDN18.2; NCT03874897). The outcomes data have been summarized in **Figure 2** and **Table 1**.

**Table 1:** Clinical trials of CAR-T cell therapies for solid tumors.

Target	Research Type	NCT Number	Tumor	Patients	Curative Effect*	Safety**	Research Institute	Reference
Glypcan-3	Clinical phase I trial	NCT02395250 NCT03146234	Hepatocellular carcinoma	13	OS at 3 years: 10.5% OS at 1 years: 42.0% OS at 6 months: 50.3%	CRS: 1 (Grade 5) ICANS: 0	Renji Hospital of Shanghai Jiaotong University, China	(16)
GD2	Clinical phase I trial	NCT02761915	Neuroblastoma	12	Not mentioned	CRS: 1 (Grade 3) ICANS: 0	Great Ormond Street Hospital for Children, UK	(17)
EGFR	Clinical phase I trial	NCT01869166	Cholangiocarcinoma and pancreatic cancer	16	Cholangiocarcinoma PFS (Median): 4 months Pancreatic Tumor PFS (Median): 3 months OS (Median): 4.9 months	CRS: 0 ICANS: 0	General Hospital of PLA, China	(18) (19)
MSLN	Clinical phase I trial	NCT03054298 NCT03323944	Malignant pleural mesothelioma, ovarian cancer, and pancreatic ductal adenocarcinoma	21	Pancreatic ductal carcinoma (6 patients) PFS: 1-5.4 months Other carcinoma (5 patients each) PFS (Median): 2.1 months SD at 28 days: 11 patients SD at 2-3 months: 3 patients	Pancreatic ductal carcinoma Not mentioned Other carcinoma CRS & ICANS: Not mentioned Death: 1 (due to liver necrosis and acute kidney injury, in 64 days)	University of Pennsylvania, USA	(20) (21)
MUC-1	Clinical phase I trial	NCT02587689	Adenocarcinoma of seminal vesicle	1	Not mentioned	Not mentioned	Soochow University, China	(22)
Her-2	Clinical phase I trial	NCT01935843	Biliary tract cancer and pancreatic cancer	11	PFS (Median): 4.8 months (1.5~8.3 months)	CRS: 1(Grade 3) ICANS: 0	General Hospital of PLA, China	(23)
PSMA	Clinical phase I trial	Not mentioned	Prostate cancer	5	PR: 2 patients	CRS: 0 ICANS: 0	Boston University School of Medicine, USA	(24)
PSMA	Clinical phase I trial	NCT03089203	Prostate cancer	13	Not mentioned	CRS: 5 (1 death with sepsis)	Perelman School of Medicine, University of Pennsylvania, USA	(25)
CEA	Clinical phase I trial	NCT02349724	Colorectal Cancer	10	Low dose CAR-T in 4 weeks: PD: 2 patients; SD: 2 patients. High dose CAR-T in 4 weeks: NE: 1 patients; SD: 5 patients.	CRS: 0 ICANS: 0	Third Military Medical University, China	(26)
Claudin18.2	Clinical phase I trial	NCT03874897	Gastric Cancer	37	OS at 6 months: 81.2% ORR at 6 months: 48.6%	CRS: 0 ICANS: 0	Peking University Cancer Hospital and Institute, China	(27)

*Short-term and long-term clinical evaluations are included in this table. OS, Overall Survival; PFS, Progress Free Survival; SD, Stable Disease; PR, Partial Responses; PD, Progressive Disease; ORR, overall response rate; NE, Not Evaluable. **CAR-T cell therapy-related adverse events beyond grade 2. Only CRS and ICANS are involved in this table. Numbers represent the number of patients unless otherwise noted.

## 4 Challenges and strategies of CAR-T cell therapy against solid tumors

In contrast to CAR T cell therapies for hematological malignancies, much less success has been achieved in solid tumor. There are several known hallmarks that contribute to the limited therapeutic efficacy in solid tumors. First, it is hard to choice an ideal target antigen, which is a major determinant of both safety and efficacy in adoptive T cell therapy (28). Second, tumor cells are surrounded by the hostile TMEs with composed with stromal cells, cytokines, and immune cells, which not only prevent T cell homing into tumor sites but also drive T cell exhaustion and dysfunction. Third, the life-threatening side effects induced by CAR T cells also present an obstacle for solid cancer treatment. Here, we discuss the above challenges in detail, as well as the current strategies to improve the therapeutic efficacy.

### 4.1 Tumor antigens fundamentally affects treatment

**Figure 3:**
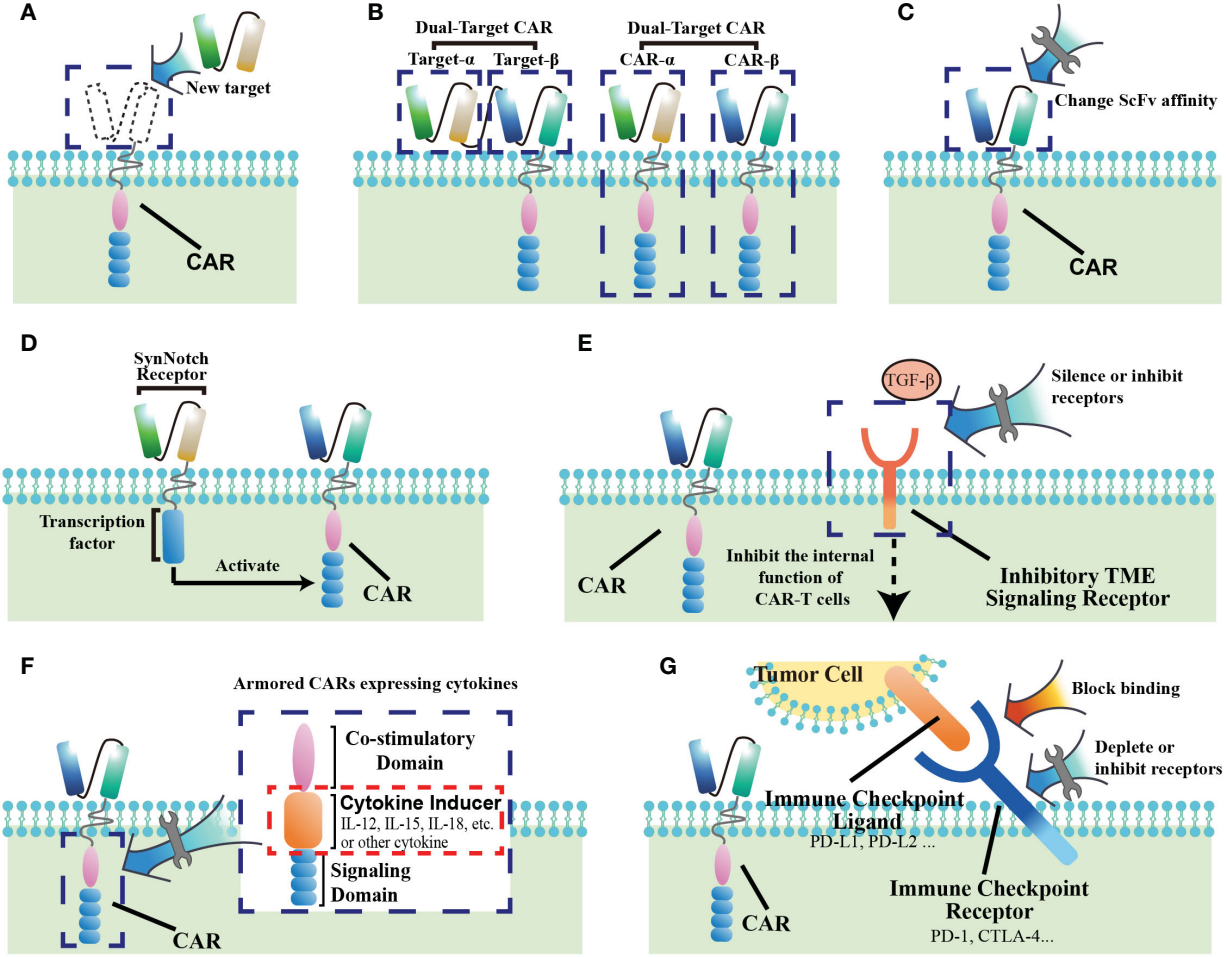
Methods of improving CAR-T cell therapy for solid tumors. **(A)** Find new targets with better specificity. **(B)** Construct dual-target CARs. **(C)** Change the affinity of the extracellular ScFv segment of CAR. **(D)** Construct SynNotch-receptor switch. **(E)** Block receptors of the inhibitory signaling from the tumor microenvironment (TME). **(F)** Armored CARs with cytokines. **(G)** Prevent CAR-T cell exhaustion through immune checkpoints.

The antigen recognition by immune cells has been identified as “lock-and-key” relationship. The selection of target antigen is a major determinant of safety and efficacy for CAR-T cell therapy. In general, tumor-specific antigens (TSAs) that are highly and homogeneously expressed within tumors are “idea” CAR targets. However, the majority of current targets are tumor-associated antigens (TAAs), which are not only enriched on tumors, but also share expression on normal tissues, albeit at a lower level. Lack of antigen specificity carries the risk of “on target/off tumor” toxicity, which could cause chronic damages to target-expressing tissues (29). Therefore, investigators have embarked on “using specific keys to open the lock”. Recently, with the development of genomic and proteomic approaches, some neoantigens with tumor specificity have been discovered (**Figure 3A**). For example, CLDN18.2 has emerged as a promising TAAs given its stable and high expression in digestive system tumors, especially gastric cancer (27). Moreover, the alternatively spliced isoforms of some oncogenes have been selected as attractive CAR targets, due that their tumor-restricted expression patterns limit “on target/off tumor” toxicity. EGFRvIII, a tumor-specific oncogenic mutation, is the most common variant of the EGFR in human cancers. An *in vivo* study demonstrated that CAR-T cells specific for EGFRvIII had efficient antitumor activity against lung cancer cells, indicating that it could be a potential therapeutic strategy to prevent recurrence and metastasis of lung cancer (30). Similarly, in a glioblastoma model, EGFRvIII-directed CAR T cells also successfully killed tumor cells *in vitro* and *in vivo* (31). Furthermore, its clinical translation in recurrent glioblastoma patients displayed potentially effective and safe that no cross-reactivity of wild-type EGFR was observed (32). Recently, a preclinical study also highlighted that CD44v6, the isoform variant 6 of CD44, could be used for CAR redirection in human lung adenocarcinoma and ovarian cancer (33). In addition to target tumor antigens directly, targeting the antigens selectively expressed in tumor vasculature is an alternative approach for CAR T therapy. As compared with healthy tissues, the glycoprotein CLEC14A selectively overexpressed on the surface of tumor vascular endothelial cells, which has been identified as a highly promising target of CAR T therapy with favorable safety profiles in the solid tumor models (34).

Solid tumors present a large degree of heterogeneity, which is a common mechanism of therapeutic resistance (35). Target-antigen expression is dynamically regulated by cancer cells in response to treatment, such that tumor cell subpopulations could escape CAR-T cell surveillance when a single targeted antigen was downregulated or completely lost. Therefore, the strategies of “using multiple keys to open the lock” are being pursued, and dual-target CARs have been designed, which either assemble two ScFvs on extracellular segment of a single CAR or express two different CARs in T cells (**Figure 3B**) (36). Recently, a CAR with antigen recognition domain of CD70 and B7-H3 in a linear fashion (also named Tandem CAR-T) had promising *in vitro* and *in vivo* results in models of lung cancer and melanoma (37). Similarly, in breast cancer model, superior antitumor responses were also observed in the engineered T cells armed with 2 CAR molecules specific for HER2 and MUC1 when compared to single targeted therapy (38). Although the clinical experience with these approaches is still limited, these data have highlighted that dual targeting may be a potential approach that not only enhance T-cell effector functions but also offset antigen escape in solid tumor.

As an artificially installed receptor on T cells, recognition of tumor expressing antigens depends on the affinity of distinct Fab fragments as well as on the density of targeted epitope, which is crucial for CAR T cell activation (39). Investigators have therefore explored “using the optimal key to open the lock”. Modulating the stability and exposure of scFvs influences CAR affinity (**Figure 3C**). ScFv optimization studies have highlighted that higher affinity is not necessarily better. In order for high levels of CAR T activation, decreasing the affinity would result in an increased requirement for higher antigen density on cells. Therefore, reduced affinity enables CAR T cell to discriminate healthy tissues with a relative low antigen expression from tumor, which could minimize CAR T therapy-related toxicities (40). Recently, with safety concerns, an antibody-based switchable CAR T system was developed, which could strictly control the activity and antigen specificity of CAR T cells by the formation of a switch-dependent immunological synapse (41). The preclinical results showed that switchable CAR-T cells against HER2 was efficacious against difficult-to-treat, patient-derived advanced pancreatic tumors, which also offered potential for safety due that CAR T activity can be modulated by switch administration (42). Additionally, in a model of NSCLC, bi-specific switch-control CAR T cells demonstrated the excellent *in vitro* efficacy and safety, which displayed antigen-specific and folate-FITC dependent reactivity against both tumor and the tumor microenvironment (43). Furthermore, another strategy to ultrasensitively discriminate antigen density is implemented by utilizing a low-affinity synthetic Notch (synNotch) receptor system, which could restrict CAR activity in tumor sites and reduce toxic cross-reaction with normal tissues. The SynNotch receptors consist of an extracellular ligand-binding domain, a transmembrane domain, and an orthogonal transcription factor (such as the transcription-activating fusion protein Gal4-VP64). After binding to its cognate ligand, the SynNotch receptor acting as a filter releases its transcription factor to induce transcription of CAR (**Figure 3D**) (44). Hernandez et al. engineered T cells with a synNotch receptor for HER2 that controlled the expression of a CAR for the same antigen, which functioned well both *in vitro* and *in vivo* with sharp discrimination between cancer cells and normal cells on the basis of HER2 expression. The results suggest that these two-step synNotch-to-CAR circuits could be a useful tool to widen the therapeutic window of engineered T cells against solid tumor (45). In another study, Tseng et al. used Logic-gated(Log) GPC3-synNotch-inducible CD147-CAR to target liver cancer cells. This CAR not only used the SynNotch receptor as a switch for tumor antigen recognition, but also had dual-target recognition specificity for GPC3 and CD147, which further limited CAR to selectively recognize GPC3^+^CD147^+^ liver cancer cells. Notably, this study also demonstrated the clinical feasibility of using SynNotch receptors to regulate CAR recognition (46).

### 4.2 Multiple components in solid tumors create immunosuppressive microenvironment

**Figure 4:**
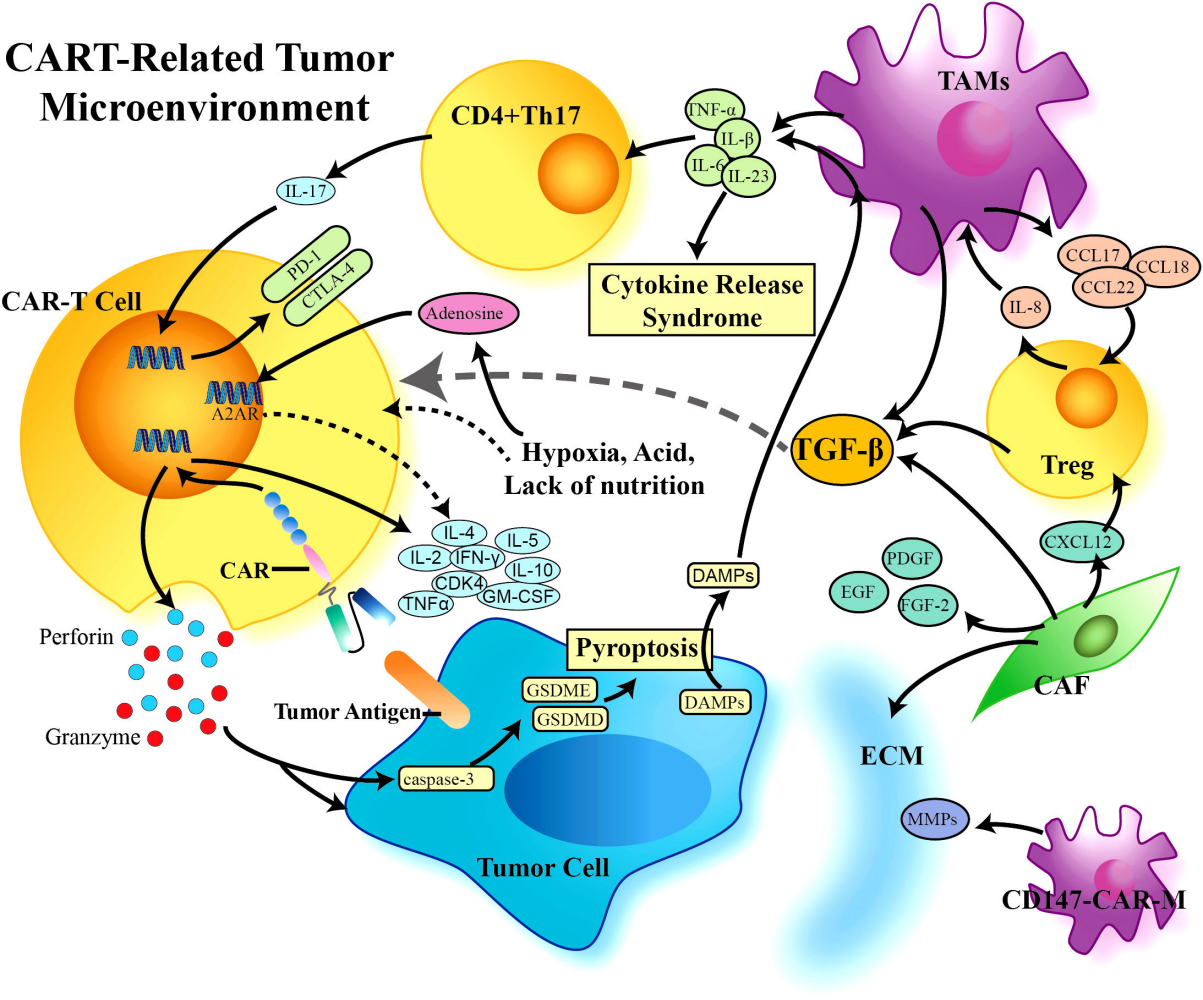
CAR-T cell-related tumor microenvironment (TME). A variety of cells participate in the formation of the tumor suppressive immune microenvironment, which limits the effect of CAR-T cells in solid tumors. TGF-β plays a central role in the TME, is produced by a variety of immunosuppressive cells, and can further negatively regulate the proliferation, differentiation, and activation of immune cells through downstream proteins and other factors. CAR, Chimeric Antigen Receptor; TGF-β, Transforming Growth Factor-β; TAMs, Tumor Associated Macrophages; Treg, Regulor T-cell; CAF, Cancer Associated Fibroblasts; ECM, Extracellular Matrix; MMPs, Matrixmetalloproteinases; DAMPs, Damage Associated Molecular Patterns.

Solid tumor cells exist in a complex tumor microenvironment, which participate in tumor progression throughout all stages of tumorigenesis (47). The TME has extensively characterized as hostile for T cells. Within the TME, the immunosuppressive cell types such as regulatory T cells (Tregs), tumor-associated macrophages (TAMs), myeloid-derived suppressor cells (MDSCs), and cancer-associated fibroblasts (CAFs) can contribute to immune evasion by inhibiting effective antitumor response of effector cells. Infiltration or polarization of these cells in human tumors has been associated with poor prognosis (48). These cells and tumor cells drive the production of tumor facilitating cytokines such as TGF-β, IL-10, and IL-4, which also promote T-cell and CAR T-cell exhaustion. The non-cellular extracellular matrix (ECM) further propagate the TME, which act as physical barriers to effectively prevent T cell infiltration. In addition, the abnormalities of the newly formed vessels have also been identified in solid tumors, which is usually leakier than normal vasculature and is therefore unable to support efficient trafficking of cytotoxic immune cells to the tumor. In general, solid tumor is considered as a special organ, which build a relatively independent system “microenvironment” to support themselves (49). Thus, the TME has a notable impact on the outcome of anticancer therapeutics. Currently, several strategies have been equipped to CAR T cells to overcome these obstacles posed by solid tumors (**Figure 4**).

#### 4.2.1 TME inhibits CAR-T activity

TAMs represent one of the main tumor-infiltrating immune cell types, which are generally categorized into classical activated M1 macrophages and alternatively activated M2 macrophages. They are two functionally contrasting subtypes and can be converted into each other upon tumor microenvironment changes. M2 type macrophage (M2) could promote IL-8 secreted by Tregs, which in turn drives the production of TGF-β, thus forming an immunosuppressive microenvironment (50). A clinical study found that increased infiltration of M2 macrophage was negatively associated with CAR T cell efficacy, which also significantly inhibited proliferation of CD4^+^ and CD8^+^ T cells (51). M-MDSCs (monocyte-related myeloid-derived suppressor cells) are currently known as a main precursor of TAMs, which are induced into TAMs by various chemokines. MDSCs are widely present in solid tumors and are characterized by their ability to suppress both innate and adaptive immune responses (52). MDSCs commonly accumulate in TME by various cytokines and chemokines, including CD47 and IL-17. Studies have shown that depleting MDSCs enhanced CAR therapy efficacy in a xenograft sarcoma when treated with CAR T cells targeting GD2 (53). Burga el al. demonstrated tumor-derived GM-CSF upregulated programmed cell death ligand 1 (PD-L1) on MDSCs in a model of liver cancer, leading to CAR T cell suppression via direct engagement of programmed cell death protein 1 (PD-1) on CAR T cells. They further found GM-CSF neutralization, MDSCs eradication or PD-L1 blockade could rescue CAR T cell function (54). Tregs are a subset of CD4^+^ T cells, which can be prohibitive of immune-mediated antitumor response. Modification of CAR T cells have specific effects on their interaction with Tregs, that CAR T cells containing both CD28 and CD3 co-stimulatory domains had superior resistance to Trges (55). In addition, immune checkpoint pathways also limit antitumor response. TAMs induce expansion of IL-17-producing CD4^+^Th17 cells to inhibit anti-tumor immunity by upregulation of PD-1, T-lymphocyte-associated protein 4 (CTLA-4), and glucocorticoid-induced TNF receptor family-related protein (GITR) (56). Activation of CAR T cells was also compromised by TAMs, which contribute to Treg activity through secretion of IL-10 as well as PD-L1 expression, suggesting that the combination of checkpoint blockage with CAR-T cells could be necessary to combat the immunosuppressive signaling in TME.

In particular, the TME is characterized by markedly elevated levels of multiple inhibitory cytokines, including TGF-β, which directly improves the function of Tregs and TAMs (**Figure 3E**). TGF-β promotes stromal cells to form a stromal phenotype, which also contributes to immune evasion by downregulated chemokine receptors. CCL22, a downstream molecule of TGF-β, is mainly produced by TAMs, which could promote Tregs recruitment, and further induce TAMs to produce TGF-β via IL-8. This positive feedback loop exacerbates the formation of inhibitory TME (57). More recently, investigators have focused on developing CARs with TGF-β signaling blockage. One of TGF-β-resistant CAR T cells was engineered via CRISPR/Cas9-mediated knockout of TGF-β receptor II, which efficiently inhibited Treg transformation, and greatly improving the function of CAR-T cells in the TGF-β-enriched TME (58). Another strategy to block TGF-β signaling is to overexpress a dominant-negative TGFβRII (TGFβRDN) in CAR T cells. Interestingly, the results of a phase I trial showed PSMA-directed CAR T cells engineered with a TGFβRDN significantly enhanced antitumor immunity in patients with castration-resistant prostate cancer (CRPC). Besides safety and feasibility in clinic, the inclusion of a TGFβRDN attenuates a common immunosuppressive barrier in the TME, with effector cytokine elaboration and CAR cell persistence, which could be a useful approach against the TME to improve outcomes (25).

In addition to the above-mentioned TAMs and Tregs, CAFs, the most prominent stromal components, have been recognized as key players in the tumor microenvironment. CAFs could induce angiogenesis, improve the oxygen and nutrient supply, and provide immunosuppressive cytokines to cancer cells, including TGF-β, epidermal growth factor (EGF), platelet-derived growth factor (PDGF), and fibroblast growth factor-2 (FGF-2) (59). CAFs heterogeneity has been identified in human breast cancer. Among the four subsets, CAF-S1 promotes immunosuppressive environment, which enhances the regulatory T cell capacity to inhibit T effector proliferation (60). More recently, fibroblast activation protein (FAP), a membrane protease, has become an attractive immunotherapeutic target, due that FAP is highly expressed on CAFs in a majority of malignant solid tumors but rarely on fibroblasts in normal tissues. The CAR T cells targeting FAP have shown potent antitumor activity in preclinical models, opening up the possibility to launch an attack on CAF-mediated immunosuppression (61).

Recent evidence has suggested that armored CARs to inducibly or constitutively secrete active cytokines or express ligands is an attractive option to create an immune-supporting milieu in the TME (**Figure 3F**) (62). IL-12, a potent inflammatory cytokine, enhances the cytotoxic ability and leads to increased secretion of IFN-γ, TNF-α and GM-CSF. In the model of hepatocellular carcinoma, CAR T cells engineered with IL-12 expression demonstrated superior antitumor efficacy with less potential side effects and decreased Treg infiltration (63). Consistently, the enhanced antitumor immune response of IL-12 armored CAR T cells was also observed in ovarian cancer (64). Another approach to overcome immunosuppressive signaling in the TME is to engineer CARs to express CD40L, with the hypothesis that CD40L mediates CD8^+^ T-cell immunity via secretion of inflammatory cytokines IL-12 and IFN-γ. CD40L-expressing CAR T-cells showed increased in vitro cytotoxicity compared to CAR T-cells alone. Furthermore, these CAR T-cells resulted in a significant survival benefit in mouse model (65). More recently, CAR T-cells engineered with IL-15 have also been developed. IL-15 is crucial for the memory differentiation and proliferation of T-cells and NK cells. Xu et al. found that coexpression of IL-15 with a CAR enhanced the *in vivo* persistence and therapeutic efficacy of CAR-NKTs (66). Due to the promising preclinical results, these armored CAR T-cells are under consideration for clinical trials. However, these cytokine-altering therapies may paradoxically potentiate some of the adverse events associated with CAR T-cells, which warrant further attention during clinical translation.

#### 4.2.2 TME restricts CAR-T cell infiltration

The hostile TME also limits the migration of CAR T cells. Efficiently trafficking into tumor tissue is a precondition to exert anti-tumor activity, which requires the interaction between chemokine secreted by tumor cell and chemokine receptor on T cells. CCR4 is the receptor of chemokine Mcp-1 and expressed on activated T cells at low levels, which plays an important role in Tregs recruitment. Currently, CCR4 antagonist has been used to treat T-cell lymphoma, demonstrating that blocking its binding enhanced CAR T cells migration to overcome the hurdles in the TME. Furthermore, the consistent results were also observed in CAR T cells specific for tumor antigen mesothelin (Msln) with co-expression of chemokine receptors CCR4 in NSCLC. As shown in a NSCLC CDX model, Msln-CCR4-CAR T cells enhanced infiltration and migration into tumor tissue, along with superior anti-tumor function and high levels of proinflammatory cytokines, including IL-2, IFN-γ, and TNF-α, suggesting that CAR T cells modified with chemokine receptor could be a potential strategy to promote T cell entry (67).

Tumor stroma, as physical barriers, also effectively prevent infiltration of CAR-T cells. Stroma, mostly composed of ECM, is more complex and denser in solid tumor than in hematological malignancy (68). The ECM is produced by all of cell types within TME, which is altered in tumor by the imbalance between ECM synthesis and secretion and changes in the levels of matrix-remodeling enzymes (69). Stroma of solid tumor inhibits CAR T cell infiltration mainly through interstitial fibrosis and high interstitial pressure. High-density ECM significantly reduced migration of immune cells and blunted cytotoxicity of lymphocytes (70). Furthermore, high-density ECM decreased the density of vessels, resulting in vessel embedding into the matrix, which are also critical for T-cell trafficking and activation (71). Therefore, many clinic trials have identified that the number of CAR T cells in tumor sites was often lower than that in peripheral blood. Thus, to overcome insufficient infiltration of CAR T cells, regional CAR T cell administration has been explored. A preclinical study with CAR-PSCA T cells on gastric cancer showed that administration pattern of CAR T cells crucially influenced their antitumor activities, in which more efficient tumor control was observed when CAR-PSCA were infused peritumorally as compared with intravenous injection, raising the concern that, besides CAR T cells themselves, some other parameters, such as administration pattern, should be considered to maximum CAR-T effector functions in clinical application (72).

Matrix metalloproteinases (MMPs), a family of calcium and zinc-dependent proteolytic enzymes, can degrade almost all components of ECM. Macrophages are an important source of MMPs. From clinical data, macrophage transplantation has been confirmed to be safe and well tolerated (73). CD147, also known as ECM metalloproteinase inducer, is essential for ECM remodeling via MMPs expression. A study in breast cancer showed that CAR-CD147 targeting HER2 macrophages, which were activated after HER2 recognition to trigger the internal signaling of CD147 and increase the expression of MMPs, did not affect proliferation of tumor cells in vitro. Interestingly, the infusion of these macrophages significantly inhibited tumor growth with HER2-positive expression in vivo, along with degrading the matrix and promoting T-cell infiltration into tumors, highlighting that targeting the ECM by engineered macrophages could be an effective strategy to augment CAR T cell infiltration into solid tumors (74). In addition to MMPs, heparanase is an enzyme to degrade heparin sulfate proteoglycan (HSPG), which is the primary component of ECM. The study verified that CAR-T cells expressing heparinase also enhanced tumor infiltration with increased antitumor activity (75). In the future, the efficacy and safety of these microenvironment-targeted therapeutic strategies remains to be further demonstrated in patients.

#### 4.2.3 TME leads to premature exhaustion of CAR-T cells

Immunosuppression in the tumor microenvironment is often based on the mutual metabolic requirements of tumor cells and immune cells. Solid tumor cells primarily use glycolysis for glucose metabolism, and this metabolic alteration renders the TME with hypoxia, acidic, deprived nutrients, and prone to oxidative stress. On the other side, activation of immune cells leads to an increased demand for glucose. This metabolic competition often limits the proliferation and effector functions of tumor-specific immune cells (76). In particular, hypoxic environment is a common mechanism to therapeutic resistance. Therefore, investigators are being explored strategies to improve CAR T cell function in low-oxygen conditions. Recently, a considerable increase of adenosine, mainly produced by endothelial cells, has been identified in hypoxic tissues. Adenosine is an important immunosuppressive factor, which has been shown to controls immune response, inflammatory tissue damage, and antitumor immunity via activation of the adenosine A_2A_ receptor (A_2A_R) in some solid tumors. A study showed that CRISPR/Cas9-mediated deletion of A_2A_R in CAR T cells significantly abrogated the immunosuppressive effects of adenosine and enhanced its *in-vivo* antitumor efficacy, with enhanced production of cytokines including IFN-γ and TNF. Importantly, no deleterious effect on memory phenotype or persistence of CAR T cells was observed (77). Another approach was to develop oxygen-sensitive CAR T cells by fusing the oxygen sensitive domain of HIF1α to CAR scaffold, which enabled very low CAR expression at normal oxygen level, but highly increased levels of CAR expression together with HIF1α in hypoxic conditions.

T cell exhaustion is a common feature of cancers. Exhausted CD8^+^ T cells (T_EX_) in cancer have limited effector function, which are characterized by the loss of cytokine production (IL-2, TNF, IFN-γ), high inhibitory receptor co-expression (PD-1, LAG3, TIGIT), altered metabolism, exhausted transcriptional profiles, and impaired proliferative potential and survival (78). T_EX_ are important clinical targets of immunotherapies. Understanding the transcriptional profiles of exhausted or exhausting T cells could promote the production of more functional CAR T cells. HMG-box transcription factor TOX has been identified as a central regulator of T_EX_, and robust TOX expression results in commitment to T_EX_ (79). Besides TOX, TOX2 as well as NR4A are also critical for the transcriptional program of CD8^+^ T cell exhaustion, all of which are targets of the calcium/calcineurin-regulated transcription factor NFAT. Within the TME, CAR T cells become exhausted and exhibit diminished ability to control the tumors. Previous study showed NR4A-deficient CAR T cells had superior efficacy at suppressing tumor growth. Another study using a CAR T cell model demonstrated that TOX and TOX2 are necessary to impose CAR T exhaustion, which were highly induced in exhausted CAR^+^ tumor-infiltrating lymphocytes (CAR TILs). Interestingly, CAR TILs deficient in both TOX and TOX2 showed increased effector functions and prolonged survival of tumor-bearing mice, and their exhausted features were obviously attenuated (80). In addition, reactive oxygen species (ROS) also function to modulate the tumor environment, affecting the various stromal cells that provide metabolic support, a blood supply and immune responses to the tumor. ROS dysregulation impairs antitumor activity of T cells (81). To render CAR T cells more resilient toward ROS, Ligtenberg et al. constructed CAR-T cells expressing catalase to improve their antioxidant capacity by metabolizing H_2_O_2_ (CAR-CAT). Compared with traditional CAR-T cells, CAR-CAT cells had a lower oxidation state with less ROS accumulation in both basal and activated states, while maintained stronger anti-tumor activity even at high H_2_O_2_ levels. These results showed that protecting CAR-T cells from exhaustion mediated by tumor-associated oxidative stress could significantly improve their efficacy (82). To date, ICB targeting PD-1 and programmed cell death ligand 1 (PD-L1) have achieved clinical success for solid tumors (83). PD-L1 is up-regulated in multiple solid tumors, which serves as a ligand for PD1 on T cells to protect tumor cells from immune control (84). The ablation of PD-1 improves T cell persistence in patients with solid tumors. Thus, blockade of the PD-1/PD-L1 axis is one of the most popular strategies to combat T cell exhaustion and restore anti-tumor immune responses mediated by CAR T cells (**Figure 3G**) (85). Besides antibody-based checkpoint blockade, several groups have explored the strategies to modify the CAR T cells with a PD1 switching receptor and a CD28 intracellular domain. Studies showed that these CAR T cells converted PD1-mediated inhibitory signals into CD28 co-stimulatory signals, which significantly augmented the efficacy of CAR T cells in solid tumors (86).To further improve the efficacy, Le et al. designed CARPD-L1z, which contained a high-affinity scFv against human PD-L1, the intracellular structure, including 4-1BB and TLR2 co-stimulatory domains, and the CD3ζ signaling domain. As compared with the forthmentioned CARs targeting PD-1 without CD 3ζ signaling domain, CARPD-L1z efficiently lysed PD-L1 positive tumor cells with enhanced cytokine secretion in vitro, which further eliminated multiple types of tumors in xenograft (87). Additionally, to reduce immune escape and increase the target specificity concomitantly, some studies developed a novel tandem CAR T cells with PD1 and an anti-MUC1 scFv, which showed more potent antitumor activity in vivo and significantly prolonged the survival time of tumor bearing mice (88), suggesting that combination checkpoint blockade-CAR-T cell therapy is likely a new immunotherapy option in solid tumor.

#### 4.3 Excessive activation of CAR T cells leads to adverse events

**Figure 5:**
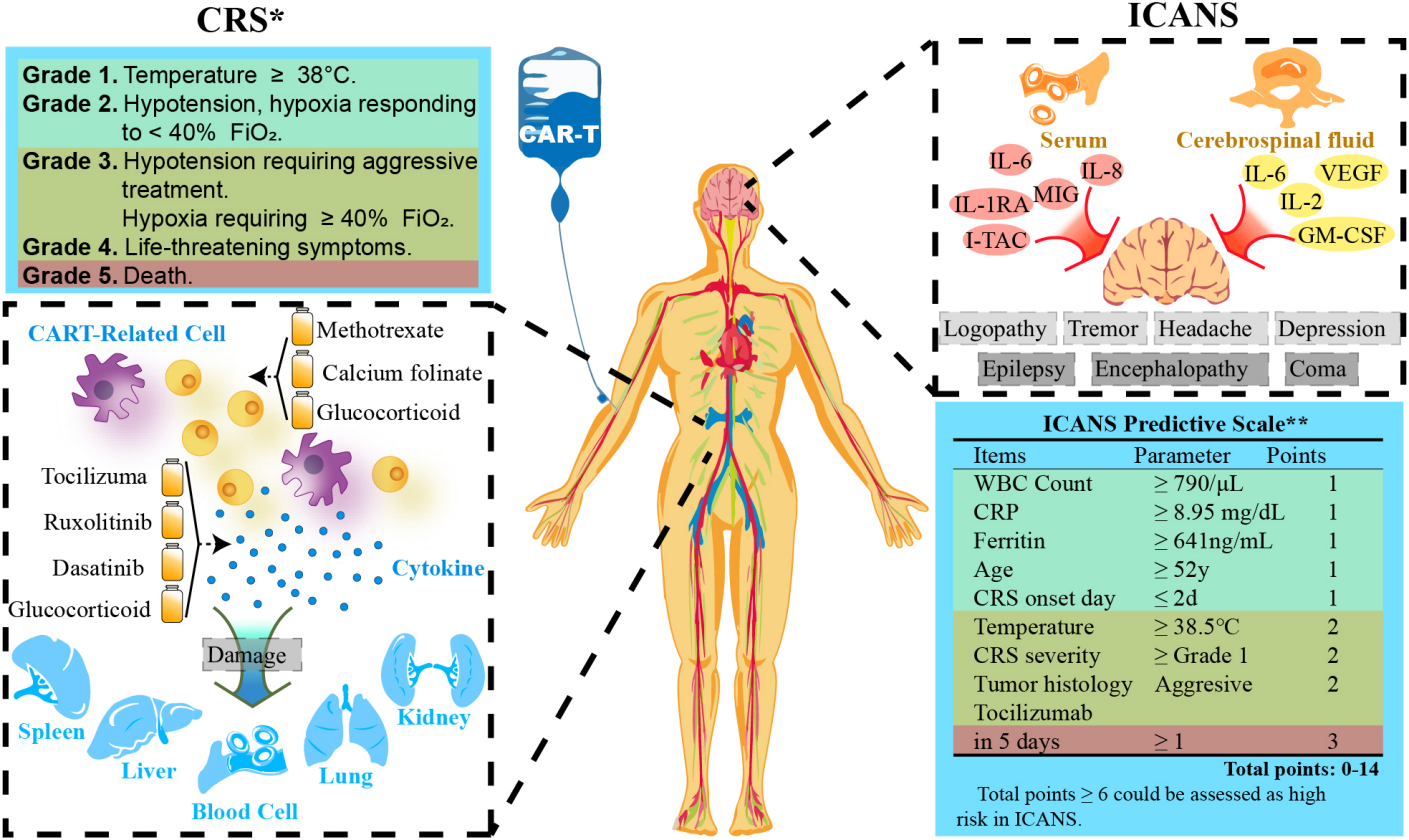
CAR-T cell therapy-related adverse events. *Grading is quoted from ASTCT (2018) (92). **Scale is referred from a study by Harvard Medical School (93). WBC, White Blood Cells; CRS, Cytokine Release Syndrome; CRP, C-reactive Protein; FiO_2_, Fraction of Inspiration O_2_.

Severe toxicities following CAR T cell administration have posed a challenge to more widespread adoption in solid tumors. Massive CAR T cell activation and severe cytokine release result in the common toxicities, including cytokine release syndrome (CRS) and neurotoxicity (89). The onset of CRS coincides near the peak of CAR T expansion and cytokine production, typically occurring during the first week after CAR T administration. After infusion, CAR T cells come into contact with target cells and release a large amount of cytokines, such as TNF-α, IL-2, IL-6, IL-8, IFN-γ, and C-reactive protein, which in turn recruit immune cells and trigger a positive feedback loop, thus consequently cause a dangerous serum concentrations of cytokines in a short period. CRS leads to ensuing systemic inflammation, capillary leakage, and coagulation cascade, which causes organ damage and is ultimately life threatening. Thus several predictive biomarkers of CRS have been identified: high tumor burden, high CAR T dose, and high peak CAR T blood counts (90). For high-risk patients, preemptive treatment could reduce the incidence of subsequent severe (Grade 3+) CRS, which indicates poor prognosis (91). Neurotoxicity, also referred immune effector cell-associated neurotoxicity syndrome (ICANS), is another unique toxicity following CAR T therapy, which usually occurs within one or 3 weeks after CAR T cell infusion. Taraseviciut et al. found that ICANS development was not only correlated with a marked accumulation of both CAR T and endogenous T cells in the cerebrospinal fluid and brain parenchyma, but also associated with pro-inflammatory cytokines and T-cell encephalitis (**Figure 5**) (94).

Mechanisms behind CAR-T-related toxicities (CARTOX) are complicated. Accumulated evidences have identified inflammatory cytokines contribute to the development of CARTOX (95), yet the source of these cytokines still remain unclear. More recently, studies highlight the importance of monocyte/macrophage in CARTOX (96), which was not related to cytokines secreted by CAR T cells themselves (97). Modulaion of macrophage function alleviated CRS severity. Direct interactions between CAR T cells and host myeloid cells – primarily macrophages – was required to produce IL-6. Moreover, Single-cell RNA-sequencing data showed that monocytes were the main source of IL-6. in patients experiencing CRS, IL-6 was found to be highly elevated, which bas been believed to primarily mediate CRS onset (98). Another study demonstrated that monocytes produced IL-1 earlier than IL-6 when cocultured with CAR T cells, suggesting that IL-1 is also linked to CRS. Recently, animal models have shown a correlation between perforin and CRS development, that perforin-deficient CAR T cells produced higher amounts of proinflammatory cytokines upon CAR-mediated in vitro activation. Additionally, cell pyroptosis, a form of inflammatory programmed cell death, has also been recognized as a major contributor to toxicity during CAR T cell therapy. Liu et al. found that CAR T cells rapidly released granzyme B in tumor cells to activate caspase 3, causing the subsequent gasdermin E (GSDME) cleavage and the release of cytokines, thus inducing CRS (99). The data further suggest GSDME as a new potential biomarker to predict the severity of CRS. Additionally, pyroptotic cells also release large amounts of DAMPs (damage-associated molecular patterns), which are endogenous immunogenic molecules released in “dangerous” situations such as tissue damage or cellular stress (100). According to the location, DAMPs have been divided into two categories, from the extracellular matrix or intracellular compartments (101). During CAR T therapy, DAMPs leakage by pyroptotic tumor cells activate macrophages and induce the release of IL-1β and IL-6, triggering CRS, highlighting the involvement of pyroptosis-DAMPs axis in CARTOX. Furthermore, some elements within the TME have been identified to correlate with CARTOX. Vascular endothelial cells appear to play an important role in CRS as reports have shown that increased levels of angiopoietin-2 and von Willebrand factor released by vascular endothelial cells were significantly associated with the severity of CRS, rendering them as biomarkers to predict prognosis of CRS (90).

Elucidating the mechanisms of CARTOX has facilitated the development of more effective treatment approaches. Current CRS management after CAR T therapy mainly relies on the blocking IL-6 with tocilizumab (recombinant humanized anti-human IL-6 receptor monoclonal antibody), which is FDA approved to treat CRS. Tocilizumab often resolves CRS symptoms within hours while the therapeutic efficacy of CAR T cells in patients was preserved. Unfortunately, tocilizumab failed to prevent neurotoxicity associated with CAR T therapy. Another way to limit toxicity of CAR T cells is to inhibit T cell receptor signal transduction via small molecule inhibitors, such as dasatinib (102). Administration of dasatinib significantly decreased levels of cytokines and protected mice from fatal CRS, which can also directly inhibit tumor-associated myeloid cells (103). Besides the forthmentioned drugs, some T-cell killing drugs, such as methotrexate and calcium folinate, have also been used to treat CRS (104). However, the toxicity on patient survival need to be careful considered because these cytokine drugs irreversibly reduce the amount of CAR T cells (105). Currently, another interesting approach is to endow CAR T cells with anti-CRS activity, which can neutralize IL-6 in the TME. Tan et al. showed that CAR T cells with a membrane bound scFv targeting IL-6 not only abrogated the symptom of CRS without compromising anti-tumor efficacy, but it had the potential to simultaneously limit the effect of TAMs (106).

## 5 Conclusions and outlook

In the latest decade, the use of cutting-edge genetic engineering techniques (especially CRISPR/Cas9) and/or combining with other modalities obviously improved the efficacy and safety of CAR T therapy for solid tumor, and raises the concerns that the landscape of CAR T in solid tumors is bright. In future research, the primary task of CAR-T cell therapy for solid tumors will be to obtain more precise and specific targets. At present, not many targets have been identified for solid tumors. Taking NSCLC as an example, most current clinical studies continue to focus on common targets such as EGFR, HER2, and their mutations. New targets may bring breakthroughs in solid tumor recognition for CAR-T cell therapy. In addition, by improving the affinity of the extracellular ScFv segment of CAR, the construction of a controllable CAR switch will further strengthen the target recognition specificity of CAR-T cells and avoid the occurrence of on-target/off-tumor effects. Moreover, the development of sequencing technology and bioinformatics will enable cells to be divided into different subtypes with similar pathological behaviors and characteristics, providing more information for the design of CAR-T targets (107). Unlike hematological tumors, the denser and more complex internal environment of solid tumors remains as barrier that must be crossed. The presence of TAMs, Tregs, CAFs, and inhibitory cytokines such as IL-10, IL-4, and TGF-β in the TME severely impacts CAR-T infiltration and survival. At the same time, the complex activation mechanism of CAR-T cells can lead to tumor immune escape and adverse events during treatment (108). Better understanding of the signaling pathways between T cells and other TME cell components, as well as the intracellular cascades associated with CAR-T cell activation and depletion, will enable greater success in the treatment of solid tumors with CAR-T cell therapy. In addition, research investigating immune cells other than T cells may lead to breakthroughs. Besides the use of CAR-M cells to break through the tumor ECM, NK cells have their own unique capabilities and are not restricted by MHC-I antigen recognition. Furthermore, CAR-NK cells can be generated from allogeneic donors (109), thus providing theoretical support for mass production of CAR cells and enabling more patients with solid tumors to benefit from CAR cell therapy. With the continuous development of oncology, the treatment of solid tumors is no longer solely based on pathological and histological type or clinical stages. More emphasis is being placed on individualized and precise treatment. Although many obstacles in the treatment of solid tumors with CAR-T cell therapy remain to be addressed, we believe that CAR-T cell therapy will become an important treatment approach for solid tumors, prolonging the survival of patients and bringing tumor treatment into a new era of precision immunotherapy.

## Author contributions

KZ and YL conceptualized, wrote, and edited the manuscript. The figures and charts were designed by YL and created by KZ. HC and FL reviewed references. SH and FC translated and edited the manuscript. All authors contributed to the article and approved the submitted version.
